# Selected comorbidities and the probability of ART switch in PWH with undetectable HIV-RNA: a retrospective analysis in Italy

**DOI:** 10.1093/jac/dkaf137

**Published:** 2025-05-12

**Authors:** Alessandro Cozzi-Lepri, Alessandro Tavelli, Lucia Taramasso, Giuseppe Lapadula, Nicoletta Bobbio, Stefania Piconi, Giovanni Guaraldi, Antonio Di Biagio, Antonella Castagna, Valentina Mazzotta, Antonella d’Arminio Monforte, Icona Foundation Study group, A d’Arminio Monforte, A Antinori, S Antinori, A Castagna, R Cauda, G Di Perri, E Girardi, R Iardino, A Lazzarin, G C Marchetti, C Mussini, E Quiros-Roldan, L Sarmati, B Suligoi, F von Schloesser, P Viale, A d’Arminio Monforte, A Antinori, A Castagna, F Ceccherini-Silberstein, A Cingolani, A Cozzi-Lepri, A Di Biagio, E Girardi, A Gori, S Lo Caputo, G Marchetti, F Maggiolo, C Mussini, M Puoti, C F Perno, C Torti, A Antinori, F Bai, A Bandera, S Bonora, A Calcagno, D Canetti, A Castagna, F Ceccherini-Silberstein, A Cervo, A Cingolani, P Cinque, A Cozzi-Lepri, A d’Arminio Monforte, A Di Biagio, R Gagliardini, A Giacomelli, E Girardi, N Gianotti, A Gori, G Guaraldi, S Lanini, G Lapadula, M Lichtner, A Lai, S Lo Caputo, G Madeddu, F Maggiolo, V Malagnino, G Marchetti, A Mondi, V Mazzotta, C Mussini, S Nozza, C F Perno, S Piconi, C Pinnetti, M Puoti, E Quiros Roldan, R Rossotti, S Rusconi, M M Santoro, A Saracino, L Sarmati, V Spagnuolo, N Squillace, V Svicher, L Taramasso, C Torti, A Vergori, A Cozzi-Lepri, S De Benedittis, I Fanti, N Lentini, M Muccio, R Pastorino, A Rodano', A Tavelli, M Cernuschi, L Cosmaro, A Perziano, V Calvino, D Russo, M Farinella, N Policek, V L Del Negro, M Augello, S Carrara, S Graziano, G Prota, S Truffa, D Vincenti, R Rovito, A Giacometti, A Costantini, V Barocci, A Saracino, C Santoro, E Milano, L Comi, C Suardi, P Viale, L Badia, S Cretella, E M Erne, A Pieri, E Quiros Roldan, E Focà, C Minardi, B Menzaghi, C Abeli, L Chessa, F Pes, P Maggi, L Alessio, G Nunnari, B M Celesia, J Vecchiet, K Falasca, A Pan, S Dal Zoppo, D Segala, M A Di Pietro, C Costa, S Lo Caputo, S Ferrara, M Bassetti, E Pontali, S Blanchi, N Bobbio, G Mazzarello, M Lichtner, L Fondaco, S Piconi, C Molteni, S Rusconi, G Canavesi, G Pellicanò, G Marchetti, S Antinori, G Rizzardini, M Puoti, A Castagna, A Bandera, V Bono, M V Cossu, A Giacomelli, R Lolatto, M C Moioli, L Pezzati, S Diotallevi, C Tincati, C Mussini, M Menozzi, P Bonfanti, G Lapadula, V Sangiovanni, I Gentile, V Esposito, N Coppola, F M Fusco, G Di Filippo, V Rizzo, N Sangiovanni, S Martini, A M Cattelan, D Leoni, A Cascio, M Trizzino, D Francisci, E Schiaroli, G Parruti, F Sozio, D Messeri, S I Bonelli, C Lazzaretti, R Corsini, A Antinori, R Cauda, C Mastroianni, L Sarmati, A Latini, A Cingolani, I Mastrorosa, S Lamonica, M Capozzi, M Camici, M Rivano Capparuccia, G Iaiani, C Stingone, L Gianserra, J Paulicelli, M M Plazzi, G d’Ettore, M Fusto, I Coledan, G Madeddu, A De Vito, M Fabbiani, F Montagnani, A Franco, R Fontana Del Vecchio, B M Pasticci, C Di Giuli, G C Orofino, G Calleri, G Di Perri, S Bonora, G Accardo, C Tascini, A Londero, G Battagin, S Nicolè, G Starnini, S Dell’Isola

**Affiliations:** CREME Centre, IGH University College London, London, UK; Icona Foundation, Milano, Italy; National PhD Programme in One Health approaches to Infectious Diseases and Life Science Research, Department of Public Health, Experimental and Forensic Medicine, University of Pavia, Pavia, Italy; Clinic of Infectious Diseases, IRCCS Policlinico San Martino Hospital, Genova, Italy; Clinic of Infectious Diseases, Fondazione IRCCS San Gerardo dei Tintori, Monza, Italy; School of Medicine, University of Milano-Bicocca, Monza, Italy; Department of Infectious Diseases, Galliera Hospital, Genova, Italy; Unit of Infectious Diseases, ASST Lecco, Lecco, Italy; Infectious Diseases Unit, Hospital Policlinico Modena, Department of Surgical and Medical Sciences, University of Modena and Reggio Emilia, Modena, Italy; Clinic of Infectious Diseases, IRCCS Policlinico San Martino Hospital, Genova, Italy; Infectious and Tropical Diseases Unit, IRCCS San Raffaele Scientific Institute, Milan, Italy; Clinical and Research Department, National Institute for Infectious Diseases Lazzaro Spallanzani IRCCS, Rome, Italy; Icona Foundation, Milano, Italy

## Abstract

**Objectives:**

To estimate the incidence of comorbidities in persons with HIV (PWH) with a stable viral load (VL) of ≤50 copies/mL and evaluate the likelihood of treatment switch (TS) according to the new development of dyslipidaemia (DP), kidney disease and a weight change that determined overweight.

**Methods:**

We carried out six case–control studies nested within the Icona Foundation Study cohort with the outcome of TS of the current regimen (due to intolerance/toxicity or simplification) and investigated the incident comorbidities. Conditional logistic regression models were employed.

**Results:**

Overall, the median age of study participants was 45 years (IQR: 36–52), 19% were female, 48% were MSM and 17% were migrants. DP was confirmed to be the most frequent incident comorbidity [138 events; incidence rate (IR) = 28.4%; 95% CI: 22.7%–34%], followed by estimated glomerular filtration rate (eGFR) deterioration and BMI elevation. None of the studied factors was associated with the risk of TS because of simplification. TS because of toxicity was predicted by incident DP [adjusted OR (aOR) = 2.49, 95% CI: 1.19–5.19, *P* = 0.02] and by a decline in eGFR of >10 mL/min/1.73 m^2^ (aOR = 1.51, 95% CI: 0.98–2.32, *P* = 0.06). The association with DP was stronger in participants who were receiving a boosted PI-based regimen at baseline (aOR = 3.38, 95% CI: 1.11–10.30, *P* = 0.03). Therapy discontinuation because of toxicity/simplification has remained common in PWH with VL of ≤50 copies/mL in recent years.

**Conclusions:**

The onset of DP and a decline in eGFR was associated with discontinuations due to toxicity. Interventions aiming to mitigate the risk of developing lipid abnormalities in PWH are likely to also reduce the number of ART changes, which can potentially affect future drug options.

## Introduction

The widespread use of ART for HIV infection has drastically reduced HIV-associated morbidity and mortality.^1–4^ However, despite the increase in life expectancy, survival rates among people with HIV (PWH) remain lower than those seen in the general population, at least in Europe, and can vary according to access and maintenance rates of care, as well as to CD4 count and viral load.^5–7^ More than half of the deaths observed in recent years among ART-experienced PWH are attributable to non-communicable diseases (NCDs).^8–11^ These include obesity, dyslipidaemia (DP), kidney disease, diabetes mellitus, hypertension, severe cardiovascular disease (CVD), bone fractures, cancer and others such as CNS disorders. The increase in the relative incidence of these conditions has led to a different approach in the clinical management of PWH, especially for ART selection in the switching setting. Specifically, it is important to evaluate whether the increased burden of NCDs has prompted clinicians to consider modifying ART towards regimens that are more tolerable and with reduced drug–drug interactions (DDIs).^12^ Indeed, there are several options that have demonstrated a good safety profile and low potential for DDIs in treatment-experienced PWH that are currently recommended by guidelines.^13,14^ PWH with comorbidities are also often referred by the primary infectious disease unit to clinics specialized in the management of each specific comorbidity.

In view of all these, it is key to document the prevalence and rate of incidence of new comorbidities in PWH, especially in the ART-switch setting, to foresee their estimated impact on daily clinical management and help inform clinical decisions regarding appropriate utilization of ART, within the various HIV treatment strategies. To date, whether newly developed comorbidities in PWH with current suppressed viral load may modify the probability of therapy modification has been rarely investigated in a large setting of PWH, regimens and type of comorbidity. It is also unclear whether the onset of comorbidities leading to a therapy modification might depend on the drug class currently received.

## Methods

The EPIdemiology and COmorbidities of people with HIV SWITCHing to current therapeutic strategies: a retrospective analysis in Italy (EPICO-SWITCH) study is a retrospective analysis of data from the Italian Cohort Naive Antiretrovirals (ICONA) Foundation cohort, an Italian nationwide observational cohort, set up in 1997, including adult PWH, ART-naive at the time of enrolment. Details of the study design and procedures have been described elsewhere.^15^ In the cohort, reasons for drug discontinuation are recorded for individual drugs by a single treating physician who completes the case-report form for that event. The main reason for discontinuing individual drugs as reported by the treating physician is used to classify discontinuations. It does happen that reported reasons do not seem to fit with the regimen currently received by the participant or with his/her history. In such cases, records are further investigated and reviewed by an expert panel who then decide to keep or modify the originally submitted reason for stopping.

**Figure 1.**
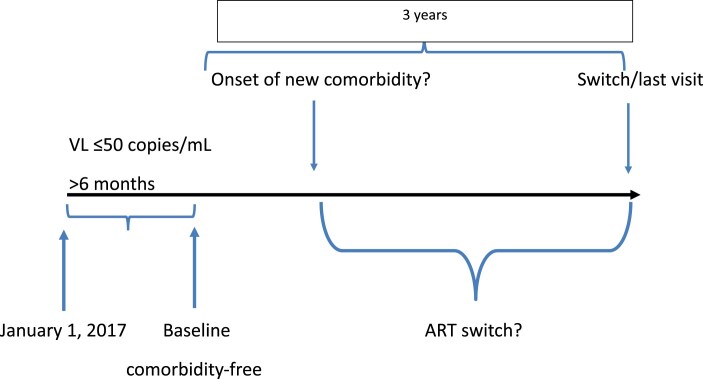
Study population and analysis design.

We constructed several matched case–control studies nested within the cohort. In order to be included in one of these case–control studies, PWH enrolled in ICONA had to satisfy the following inclusion criteria: (i) they had to have a period of >6 months with an HIV-RNA of ≤50 copies/mL on ART (the date of the second value ≤50 copies/mL was the baseline date for the survival analysis); (ii) baseline had to be after 1 January 2017 regardless of the date of first achieving viral suppression; (iii) they had to have ≥1 clinical visit post baseline [Figure 1, Figure S1 (available as Supplementary data at *JAC* Online)]; and (iv) they had to be free from the NCDs of interest at baseline [e.g. in the case–control study focusing on estimated glomerular filtration rate (eGFR) decline, PWH could only be included if they had a eGFR of >60 mL/min/1.73 m^2^ at baseline]. The year 2017 was used to include PWH with successful viral suppression obtained with regimens currently recommended by guidelines. However, all participants satisfying the criteria (i–iv) above have been included regardless of the number of therapy lines previously received and of the exact regimen received at baseline. We used the data of the ICONA database updated to 31 July 2024.

### Outcomes

We defined as cases: (i) discontinuation of ≥1 drug in the regimen received at baseline due to intolerance/toxicity; and (ii) discontinuation of ≥1 drug in the regimen received at baseline due to simplification. Simplification has been defined following the HIV Cohorts Data Exchange Protocol (HICDEP) coding, which includes reasons ranging from ‘the treatment is too complex’ to ‘drug-to-drug interactions’ and ‘change in eligibility criteria or treatment protocol’. Because some of these switches occur after prolonged suppression, to have sufficient statistical power we modelled the 3 year probability of discontinuation as a binary outcome.

Controls were PWH enrolled in ICONA matched for sex at birth, age (±5 years) and time from baseline to the date of therapy switch. We selected a maximum of three controls for a case in the matched sets. We only included complete matched sets with one case and at least one matched control in the analyses.

### Exposures

The main exposure of interest was the new onset after baseline of one of the three conditions of interest, which were defined as follows: (i) becoming overweight (OW)^16,17^ (first time BMI ≥ 26 kg/m^2^ from a baseline BMI ≤ 25 kg/m^2^, or incident increase in BMI of >3 kg/m^2^, regardless of baseline); (ii) newly developing DP [new initiation of lipid-lowering drug therapy or a total cholesterol (TC)/HDL ratio rise to >5 (males) or >4.4 (females)];^18^ and (iii) newly developing kidney disease (KD)^19^ [eGFR (CKD-Epi formula) < 60 mL/min/1.73 m^2^ starting from a baseline value of ≥60 mL/min/1.73m^2^, or incident decline in eGFR (CKD-Epi formula) of >10 mL/min/1.73 m^2^].

These three exposures were chosen over others because they are very common conditions in PWH (and therefore the statistical analysis would be adequately powered) and because all three can be well documented in our cohort. In sensitivity analysis we defined onset of KD as confirmed decline to <60 mL/min/1.73 m^2^ (two consecutive values). We also used an alternative definition for BMI using the standardized WHO definition of BMI ≥ 25 kg/m^2^ from a baseline BMI of ≤24 kg/m^2^.

### Potential effect measure modifiers

The evaluation of the possible effect of specific drugs or regimens, as stipulated at the time of writing the protocol, was beyond the scope of this work. Nevertheless, we had specific pre-specified hypotheses to test. For example, that the association between some exposures and outcome might vary by the class of anchor drug received at baseline [e.g. triple boosted PI-based and DP; integrase strand transfer inhibitor (INSTI)-based and both weight gain and eGFR].

### Statistical analysis

First, to estimate the incidence of comorbidities (the exposure in the case–control study), survival analyses have been conducted of the time from baseline to occurrence of a new comorbidity. The 2 year incidence of new onset of comorbidity was estimated using the Kaplan–Meier method and reported. Demographics, HIV-related, and clinical characteristics at baseline were described for the case–control study with the largest sample size and stratified by the incident comorbid exposure (outcome: switch due to simplifications and incident eGFR decline to <60 mL/min/1.73 m^2^ as the main exposure). In the survival analysis underlying the case–control studies, time accrued from baseline to the time of therapy modification (due to intolerance/toxicity or simplification) and was censored if there was evidence of viral rebound (VR) of >50 copies/mL or at the last clinical follow-up, whichever occurred first. We censored participants’ follow-up at the time of VR > 50 copies/mL because we were only interested in switches occurring when viral load was still suppressed.

We calculated the prevalence of the exposure in cases and controls and used conditional univariable and multivariable logistic regression models to evaluate the association between incident comorbidities and the probability of a therapy change. Multivariable models were controlled for time-fixed covariates measured at baseline. In a sensitivity analysis we furthered controlled for class of the anchor drug received by participants at index date. Of note, this is an exposure-wide analysis, so the set of confounding variables included was slightly different in each of the case–control studies as it had been tailored to the specific time-varying exposure of interest. The whole set of confounders included: year of baseline, age (fitted as continuous), sex at birth, obesity (defined as BMI > 30 kg/m^2^), alcohol use, nationality (Italian-born versus foreign) and a diagnosis of diabetes, AIDS and CD4 count at baseline and have been identified based on subject-matter knowledge applying the disjunctive cause criterion.^20^ We did not control in the model for time-varying potential confounders, except for the current regimen (in a sensitivity analysis). Because the set of confounders was identified at the outset, the data extractions for the case–control analysis only include participants with complete data for these factors, although the rate of missing data in the ICONA cohort for this key confounding factors is <5%. Statistical interaction between the exposure variable (new onset of each comorbidity) and the anchor drug in the baseline ART regimen was formally tested by including an interaction term in the logistic regression models. If there was evidence for interaction, then results were stratified by anchor drug class received at baseline.

Breakdown of the main reason for discontinuations was plotted by means of pie charts. The distribution of type of therapies, in terms of number of drugs [ two-drug regimen (2DR) versus three-drug regimen (3DR)], and class of the anchor drug used, which were started after the discontinuation of the baseline regimen, was also described. Of note, per protocol for this analysis, we had stipulated not to perform analyses involving individual drugs and these will be not shown as part of this work.

All statistical analyses were performed using SAS (version 9.4, SAS Institute, Cary, NC, USA). All *P* values presented are two-sided and we used 0.05 as the threshold for type I error.

### Ethics

The ICONA Foundation study was approved by the local Ethics Committees of participating clinical sites. All patients signed a consent form for study participation and processing of data in accordance with the ethical standards of the committee on human experimentation and the Declaration of Helsinki (last amended in October 2013).

## Results

### Study population

Because of the different inclusion criteria, tailored to the specific outcome and comorbidities profile at baseline, each separate case–control study has a slightly different number of participants included (Figure S1, Table S1). Here we describe the main characteristics of the largest of these studies in which PWH discontinued because of simplification and a history of DP could be reconstructed for them (with a total sample size of 2992, of whom 1032 were cases and 1960 were matched controls—population d3 in Figure S1). The cases of the other nested case–control studies are essentially subsets of the cases described here. Briefly, the number of stops due to simplification ranged between 680 (for BMI) and 1032 (for eGFR), while those due to toxicity ranged between 219 (for BMI) to 307 (for eGFR) stops, across case–control studies, depending on the exact exposure examined.

In detail, of the total 2992 PWH included, 143 (5%) experienced a decline in eGFR to <60 mL/min/1.73 m^2^ and 1032 (34%) modified therapy because of simplification after baseline. It was not possible to obtain two controls in each of the matched sets and we ended up with a total of 928 cases with two controls (total of 1856 controls) and 104 cases with only one control for a total of 1856 + 104 = 1960 controls (Figure S1).

Overall, median age was 45 years (IQR: 36–52), 19% were female, 48% acquired HIV through MSM contacts and 17% were of non-Italian nationality. A small proportion (13%) had been previously diagnosed with AIDS, and the vast majority (88%) were receiving a regimen including ≥3 antiretrovirals at baseline. Among all therapies, the anchor class drug received at baseline was a boosted PI (PI/b) in only 487 participants (23%) . At baseline, as per the inclusion criterion, they had an HIV-RNA of ≤50 copies/mL for an average of 7.8 months (IQR: 6.6–10.1). The median time from onset of the exposure to discontinuation was approximately 8–10 months, depending on exact exposure and outcome. For example, when the outcome was the switch due to simplification, the eGFR of <60 mL/min/1.73 m^2^ was recorded 10 months (IQR: 6–17) and DP 9 months (IQR: 6–14) before the event; when the outcome was the switch due to toxicity, the equivalent lengths of time were 9 (IQR: 6–17) and 8 (IQR: 6–16) months, respectively (Table 1).

**Table 1.** Characteristics of PLWH included in the case–control study with switch due to simplification and eGFR data

	Incident eGFR decrease to <60 mL/min/1.73 m^2^			
Characteristics at baseline^a^	Yes	No	*P* value^b^	Total
	*n* = 143	*n* = 2849		*n* = 2992
Gender, *n* (%)			0.084	
Female	35 (24.5)	532 (18.7)		567 (19.0)
Mode of HIV transmission, *n* (%)			0.110	
PWID	15 (10.5)	253 (9.0)		268 (9.0)
MSM	59 (41.3)	1371 (48.6)		1430 (48.3)
Heterosexual contacts	65 (45.5)	1046 (36.7)		1111 (37.1)
Other/unknown	4 (2.8)	149 (5.3)		153 (5.2)
Nationality, *n* (%)			0.182	
Not Italian	19 (13.3)	502 (17.6)		521 (17.4)
AIDS diagnosis, *n* (%)			0.006	
Yes	29 (20.3)	353 (12.4)		382 (12.8)
CVD diagnosis, *n* (%)			0.008	
Yes	6 (4.2)	40 (1.4)		46 (1.5)
HBsAg, *n* (%)			0.317	
Negative	133 (93.0)	2562 (89.9)		2695 (90.1)
Positive	3 (2.1)	48 (1.7)		51 (1.7)
Not tested	7 (4.9)	239 (8.4)		246 (8.2)
HCVAb, *n* (%)			0.024	
Negative	113 (79.0)	2348 (82.4)		2461 (82.3)
Positive	24 (16.8)	293 (10.3)		317 (10.6)
Not tested	6 (4.2)	208 (7.3)		214 (7.2)
Calendar year of baseline^c^			0.423	
Median (IQR)	2018 (2017–19)	2018 (2017–19)	0.524	2018 (2017–19)
2017, *n* (%)	69 (48.3)	1273 (44.7)		1342 (44.9)
2018, *n* (%)	35 (24.5)	802 (28.2)		837 (28.0)
2019, *n* (%)	17 (11.9)	303 (10.6)		320 (10.7)
2020, *n* (%)	6 (4.2)	174 (6.1)		180 (6.0)
2021, *n* (%)	11 (7.7)	127 (4.5)		138 (4.6)
2022–24, *n* (%)	5 (3.5)	170 (6.0)		175 (5.8)
Age, years			<0.001	
Median (IQR)	55 (48–61)	44 (36–51)		45 (36–52)
CD4 count, cells/mm^3^				
Median (IQR)	643 (446–865)	703 (518–920)	0.020	701 (515–919)
≤200 cells/mm^3^, *n* (%)	3 (2.1)	63 (2.2)	0.928	66 (2.2)
CD4 count nadir, cells/mm^3^			<0.001	
Median (IQR)	222 (96–363)	308 (169–449)		306 (164–446)
CD8 count, cells/mm^3^			0.805	
Median (IQR)	831 (588–1125)	840 (619–1116)		840 (617–1116)
eGFR (CKD_Epi formula), mL/min/1.73m^2^				
Median (IQR)	68.17 (63.79–75.26)	91.83 (80.65–103.4)	<0.001	90.80 (78.97–102.8)
Below 60, *n* (%)	0 (0.0)	0 (0.0)		0 (0.0)
Site geographical location, *n* (%)			0.064	
North	79 (55.2)	1631 (57.2)		1710 (57.2)
Centre	58 (40.6)	962 (33.8)		1020 (34.1)
South	6 (4.2)	256 (9.0)		262 (8.8)
Diabetes, *n* (%)			<0.001	
Yes	26 (18.2)	263 (9.2)		289 (9.7)
Smoking, *n* (%)			0.509	
No	74 (51.7)	1354 (47.5)		1428 (47.7)
Yes	54 (37.8)	1119 (39.3)		1173 (39.2)
Unknown	15 (10.5)	376 (13.2)		391 (13.1)
Total cholesterol, mg/dL			0.100	
Median (IQR)	189 (164–219)	184 (159–211)		184 (159–212)
HDL cholesterol, mg/dL			0.413	
Median (IQR)	46 (40–59)	46 (39–56)		46 (39–56)
Use of statins, *n* (%)			<0.001	
Yes	29 (20.3)	234 (8.2)		263 (8.8)
Use of blood pressure lowering drugs, *n* (%)			<0.001	
Yes	30 (21.0)	244 (8.6)		274 (9.2)
Time from HIV diagnosis to baseline, months			<0.001	
Median (IQR)	92 (44–183)	60 (26–120)		62 (27–122)
Blood glucose, mg/dL			0.015	
Median (IQR)	89 (81–103)	87 (79–95)		87 (80–95)
Type of regimen in episode, *n* (%)			0.114	
Dual	29 (21.2)	304 (11.0)		333 (11.5)
Triple	106 (77.4)	2427 (87.7)		2533 (87.2)
Four or more drugs	2 (1.5)	36 (1.3)		38 (1.3)
PI-based	33 (23.1)	645 (22.6)	0.903	678 (22.7)
Education, *n* (%)			0.007	
Primary school	6 (4.2)	105 (3.7)		111 (3.7)
Secondary school	32 (22.4)	518 (18.2)		550 (18.4)
College	53 (37.1)	859 (30.2)		912 (30.5)
University	14 (9.8)	389 (13.7)		403 (13.5)
Other/unknown	38 (26.6)	978 (34.3)		1016 (34.0)
Employment, *n* (%)			0.001	
Unemployed	18 (14.1)	320 (13.5)		338 (13.5)
Employed	66 (51.6)	1252 (52.7)		1318 (52.6)
Self-employed	29 (22.7)	453 (19.1)		482 (19.2)
Occasional	3 (2.3)	75 (3.2)		78 (3.1)
Student	1 (0.8)	111 (4.7)		112 (4.5)
Retired	7 (5.5)	31 (1.3)		38 (1.5)
Invalid	0 (0.0)	6 (0.3)		6 (0.2)
Housewife	3 (2.3)	68 (2.9)		71 (2.8)
Other/unknown	1 (0.8)	60 (2.5)		61 (2.4)
Duration of VL suppression, months			0.450	
Median (IQR)	8.0 (6.6–10.3)	7.8 (6.7–10.1)		7.8 (6.6–10.1)

HCVAb, HCV antibody.

^a^Date of the switch (cases) or of last VL.

^b^Chi-squared or Mann–Whitney test as appropriate.

^c^Sustained VL suppression after January 2017.

#### Incidence of exposure

The rate of incidence of studied comorbidities by subcohort over the first 2 years of follow-up are shown in Figure S2(a, b and c). As expected, DP was confirmed to be the most frequent incident comorbidity (138 events; IR = 28.4% by 48 months; 95% CI: 22.7%–34%, followed by eGFR deterioration (86 events; IR = 18.4% by 48 months; 95% CI: 13.7%–23% and BMI elevation (45 events; IR = 13.7% by 48 months; 95% CI: 98.9%–18.4%).

### Discontinuation due to simplification outcome

None of the exposures of interest showed an association with the probability of modifying therapy because of simplification (Table 2). Indeed, all adjusted ORs (aORs) were close to 1 with narrow CIs so that the data overall carried no evidence against the null hypothesis of no association.

**Table 2.** OR of ART modification due to simplification according to incident comorbidities from fitting a conditional logistic regression model with time-fixed confounding factors measured at baseline

	Unadjusted and adjusted ORs of discontinuation due to simplification					
	Cases	Controls	Unadjusted^a^		Adjusted^a,b,c,d^	
	*n* (%)	*n* (%)	OR (95% CI)	*P* value	aOR (95% CI)	*P* value
New onset of DP				0.846		0.928
No	755 (90.0)	1246 (89.6)	1		1	
Yes	84 (10.0)	145 (10.4)	0.97 (0.73–1.29)		1.02^b^ (0.69–1.49)	
New onset of eGFR < 60 mL/min/1.73 m^2^				0.924		0.876
No	980 (95.0)	1869 (95.4)	1		1	
Yes	52 (5.0)	91 (4.6)	1.02 (0.70–1.48)		1.03^c^ (0.71–1.50)	
Decline in eGFR of >10 mL/min/1.73 m^2^				0.284		0.276
No	860 (83.3)	1661 (84.7)	1		1	
Yes	172 (16.7)	299 (15.3)	1.12 (0.91–1.40)		1.13^c^ (0.91–1.40)	
New onset of BMI > 26 kg/m^2^				0.699		0.747
No	640 (94.1)	1215 (94.8)	1		1	
Yes	40 (5.9)	67 (5.2)	1.10 (0.67–1.83)		1.12^d^ (0.56–2.26)	
Increase in BMI > 3 kg/m^2^				0.936		0.902
No	250 (92.9)	411 (92.2)	1		1	
Yes	19 (7.1)	35 (7.8)	1.04 (0.41–2.66)		0.95^d^ (0.45–2.04)	

	Unadjusted and adjusted ORs of discontinuation due to toxicity/intolerance					
	Cases	Controls	Unadjusted^a^	Adjusted^a,b,c,d^		
	*n* (%)	*n* (%)	OR (95% CI)	*P* value	aOR (95% CI)	*P* value
New onset of DP				0.002		0.015
No	219 (86.9)	510 (93.8)	1		1	
Yes	33 (13.1)	34 (6.3)	2.43 (1.37–4.32)		2.49^b^ (1.19–5.19)	
New onset of eGFR < 60 mL/min/1.73 m^2^				0.350		0.325
No	296 (96.4)	605 (95.7)	1		1	
Yes	11 (3.6)	27 (4.3)	0.67 (0.30–1.54)		0.66^c^ (0.28–1.52)	
Decline in eGFR > 10 mL/min/1.73 m^2^				0.033		0.064
No	257 (83.7)	565 (89.4)	1		1	
Yes	50 (16.3)	67 (10.6)	1.59 (1.04–2.44)		1.51^c^ (0.98–2.32)	
New onset of BMI > 26 kg/m^2^				0.585		0.708
No	211 (96.3)	362 (96.3)	1		1	
Yes	8 (3.7)	14 (3.7)	0.77 (0.30–1.98)		0.83^d^ (0.32–2.18)	
Increase in BMI > 3 kg/m^2^				0.866		0.807
No	45 (88.2)	94 (92.2)	1		1	
Yes	6 (11.8)	8 (7.8)	1.19 (0.16–8.60)		1.16^d^ (0.34–3.93)	

^a^Matched for sex at birth and age.

^b^Adjusted for year of baseline, age, sex at birth, obesity, alcohol use and nationality, CD4 base and AIDS.

^c^Adjusted for year of baseline, age, diabetes and sex at birth, CD4 base and AIDS.

^d^Adjusted for year of baseline, age, sex at birth and nationality, CD4 base and AIDS.

### Discontinuation due to toxicity outcome

**Figure 2.**
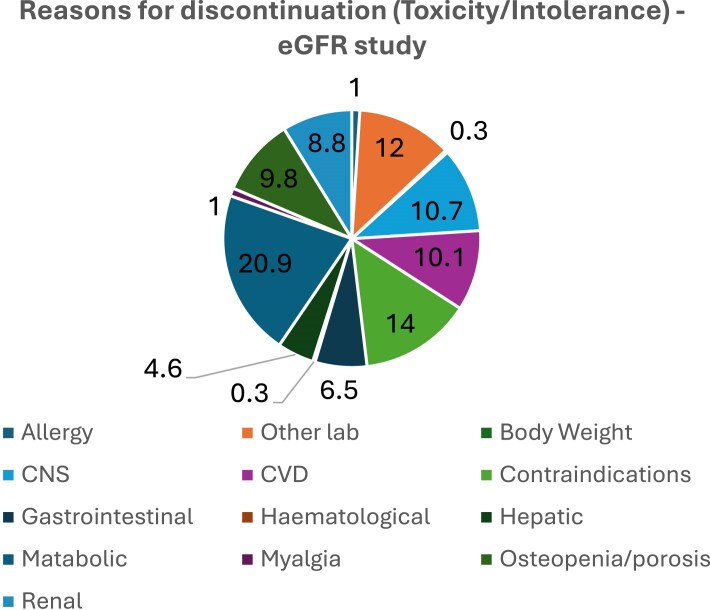
Distribution of the main reasons for modification due to toxicity/intolerance (eGFR exposure study).

Figure 2 shows the breakdown of the type of discontinuations due to toxicity in the case–control study evaluating the association between incidence of eGFR deterioration and risk of discontinuation. The most frequent reason for discontinuing was metabolic complications (i.e. DP with or with diabetes or other metabolic syndromes) (21%), followed by other laboratory parameters abnormalities (12%) accounting for one-third of the total discontinuations. The breakdown was similar in the case–control study evaluating the ART modification due to toxicity following a new onset of DP (Figure S3). After controlling for time-fixed baseline confounding, a new onset of DP was associated with a >2.5-fold higher probability of ART switch due to toxicity [adjusted HR (aHR) = 2.49, 95% CI: 1.19–5.19, *P* = 0.02 Table 2]. Results were also similar after further controlling for type of regimen currently received (Table S2). This association was even stronger in the subset analysis restricted to only participants who were receiving PI-based regimens at baseline (aOR = 3.38, 95% CI: 1.11–10.30, *P* = 0.03).

An incident decline in eGFR of >10 mL/min/1.73 m^2^ was compatible with a reduction in risk of discontinuation due to toxicity of only 3% but also with a >2-fold increased risk after controlling for confounding (aOR = 1.51, 95% CI: 0.98–2.32, *P* = 0.06). Also, this association was attenuated after controlling for type of current regimen received (Table S2) but much stronger in the subset of participants who were receiving INSTI-based regimens at baseline (aHR = 2.84; 95% CI: 1.00–8.07, Table S3). In contrast, regarding the association between an incident decline to a value of <60 mL/min/1.73 m^2^ and risk of ART discontinuation, results were inconclusive, regardless of the KD definition used (single versus confirmed value, Table S4).

In contrast, we found no evidence that ART switch due to toxicity was associated with an observed change in body weight that determined overweight, both in the overall analysis, when using the alternative WHO definition (Table S5) and after restricting to PWH who were receiving INSTI-based therapy at baseline (no evidence for an interaction, not shown).

**Figure 3.**
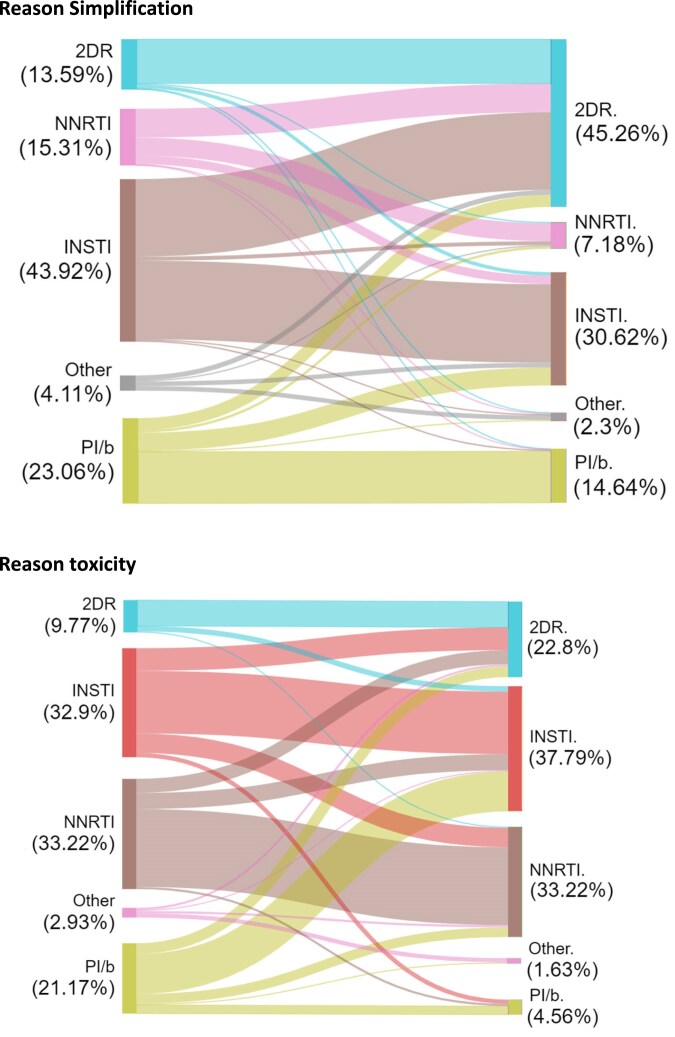
Class of the anchor drug used at baseline and after ART modification (only cases after eGFR decline).

Finally, Figure 3 shows the distribution of the class of the anchor drug included in the modified regimen following either a discontinuation due to intolerance/toxicity or simplification and according to the class used at baseline. Thus, for example, after a discontinuation for simplification the proportion of participants who were receiving a 2DR increased from 14% to 45% with most of the PWH switching from an INSTI-based 3DR regimen to a 2DR regimen. Of note, modifications to 2DR regimens were much rarer following a discontinuation due to toxicity. In contrast, after a discontinuation because of toxicity, as expected, the most frequently observed migration was from triple PI/b-based regimens to either triple INSTI-based or 2DR regimens (Figure 3). Similar switching patterns were seen in the case–control study focusing on DP (Figure S4).

## Discussion

Our analysis shows that, in the setting of PWH with a suppressed viral load evaluated during recent person-time at risk (after January 2017), ART modification due to simplification and toxicity remains common. This is remarkable considering that we included participants who at baseline were free from specific comorbidities under study. Among the comorbidities considered, incident DP and discontinuations due to metabolic complications were the most common events.

Furthermore, the development of DP over follow-up appeared to be associated with a >2.5-fold greater probability of ART switch due to toxicity, which was even stronger in participants receiving PI/b-based regimens. Moreover, there was some evidence that an incident decline in eGFR of >10 mL/min/1.73 m^2^ was also a factor associated with the probability of ART switch due to toxicity (leading to a 50% increase in risk), although with some uncertainty around this estimate. Of note, we did not see any association between an increase in body weight (that determined overweight) and the risk of stopping due to simplification. This latter finding is interesting as it seems to support the notion that changes of ART due to simplification in current clinical practice are pro-active switches determined by convenience (pill reduction, migration to a single-tablet regimen) rather than failure of the treatment because of the onset of specific comorbidities.

Studies of predictors of ART discontinuation among populations with a suppressed viral load are limited. Most of the studies so far have been conducted in ART-naive populations or in PWH who have started a second line-ART after failure of the first one.^21,22^ Many studies also focused on discontinuations tout court without distinguishing by the reason for discontinuation. In terms of determinants of discontinuation, the most frequently evaluated factors were HIV-RNA levels, individual drugs or drug classes and the modality of HIV transmission, with a focus on persons who inject drugs (PWID).^22,23^ In addition, most of these studies were done before the widespread use of INSTIs, and toxicity or intolerance was the most frequent reason for discontinuation reported by the treating physicians instead of simplifications.^21,22,24^

To our knowledge there are no studies that have specifically investigated the role of time-varying DP as a possible determinant of ART modification in the HIV setting. Our analysis indicates that experiencing *ex novo* DP was associated with a higher probability of ART switch; of interest and consistent with this finding, for one-third of the discontinuations the treating physician reported metabolic toxicity or elevation of laboratory parameters as the main reason for discontinuing ART. Because of the observed direct association between levels of LDL cholesterol (LDL-C) and risk of coronary heart disease (CHD) and mortality in the general population,^25–28^ in PWH with altered cholesterol levels, HIV treatment guidelines suggest, in the first instance, to advise lifestyle modification interventions including diet, exercise and smoking cessation.^29^ If the individual is unable to reach lipid targets with lifestyle modification interventions, lipid-lowering drugs should be started, or modification of ART should be considered.^30^ Our definition of DP did incorporate the initiation of lipid-lowering drugs, and our results suggest that the probability of ART switch might be altered by the current use of these drugs, especially in participants who were receiving a PI/b-based regimen. This is in line with what was expected as most PIs and boosting agents are known to increase LDL-C and triglycerides,^31^ which may have guided the clinical decision to change the anchor drug.^32^

Moreover, comorbidities associated with obesity make weight gain and metabolic changes a major consideration in studies evaluating safety of ART regimens. To date, the role of individual antiretrovirals or classes of weight gain remains unclear, given the multifactorial nature of weight change, and there are currently no guideline recommendations regarding switching to alternative regimens based on the evidence that they might slow, halt or reverse weight gain or metabolic changes. There are also widely heterogeneous cultural and ethnic views on what is a ‘normal’ weight.^33^ Furthermore, weight gain must be related to baseline weight, as individuals with HIV advanced disease are underweight in most cases, due to chronic debilitating conditions and/or AIDS (return to health condition thanks to ART). Many people welcome weight gain after treatment initiation and indicate that they would prefer to remain on the same drugs, raising issues around autonomy and people empowerment. BMI is commonly used to assess obesity, despite a poor correlation with actual fat mass. Although it is easy to measure, and is stratified into standardized categories, BMI is known to be weakly correlated with clinical outcomes.^34^ Our results likely reflect this status of uncertainty in the HIV community as BMI elevation was not associated with an increased probability of switching ART, regardless of the reason. Of interest, this was confirmed even in the subset of participants currently receiving INSTI-based regimens, despite there being some published evidence that weight gain might be worse with this drug class.^17,35^ Another, somewhat expected, but key result of our analysis was the fact that after a discontinuation due to simplification, a migration to 2DR regimens was frequently observed while most participants switched away from PI/b-based regimens after discontinuations due to toxicity. A very recent French study reported that participants with obesity were more likely to switch to doravirine-based regimens compared with other regimens not containing doravirine.^36^

Finally, our data also carried evidence of an effect of eGFR decline on probability of ART modification, especially in participants who were receiving INSTI-based regimens at baseline. This is interesting as clinical trials showed that the increase in serum creatinine with INSTI is not associated with a reduction in renal function.^37^ We detected this association even though, most of the switch from tenofovir disoproxil fumarate to tenofovir alafenamide-based regimens are likely to have occurred in Italy before the time zero for this analysis. This finding is conflicting with the results of another study conducted in the CNICS cohort, which reported that among PWH who initiated ART, including some who started TDF-based ART, kidney dysfunction was not a major factor leading to regimen modification.^38^ Of interest, we investigated both the effect of a decline in eGFR below a fixed threshold and the effect of detecting a decline of >10 mL/min/1.73 m^2^, regardless of the current absolute value, and we detected an association only for the latter, suggesting that unexpected large variations in eGFR may induce the clinician to modify ART.

Before drawing firm conclusions, some limitations need to be mentioned. First, the analysis is likely to be underpowered for some of the associations (e.g. eGFR decline < 60 mL/min/1.73 m^2^) and for detecting clinically important interactions. First, we also looked at incident diabetes as a possible additional NCD of interest and we found only two new incident diagnoses in PWH in the whole ICONA cohort who were free from diabetes at baseline, so this part of the analysis was unfeasible and eventually removed from the current work. Second, our analysis assumes that there is no unmeasured confounding, the models are correctly specified, and, above all, there are no time-varying confounding factors affected by previous exposure. This is particularly critical for the model with BMI and DP as an exposure, as diet and exercise are key factors associated with weight, cholesterol levels and potentially also with the risk of stopping ART. However, the assumption regarding time-varying confounders seems reasonable for most of the considered exposures. In the case of the association with DP, initiation of lipid-lowering strategies is already included in the definition of the exposure. We also performed a sensitivity analysis after controlling for anchor drug class included in the current ART regimen, carrying similar results. Finally, we selected *a priori* a set of comorbidities of interest, based on clinical judgement, data availability and statistical considerations but this is only a limited number of NCDs affecting the life and treatment management of PWH. The data collected in ICONA lack sufficient accuracy to investigate the role of some of these other important diseases (e.g. CNS-related disorders). Last, although we used established definitions for the comorbidities, these are not fully standardized, and results may have been different when using alternative definitions. Some choices were forced by the low incidence of specific events. For example, to increase statistical power, the definition of KD in the man analysis was based on a single value of eGFR of <60 mL/min/1.73 m^2^ instead of two consecutive values, as is often used. However, results were similar regardless of the exact definition used. An increase of 3 kg regardless of baseline is quite unspecific and may not be a source of worry for clinicians in patients who are underweight. Finally, our participants were all enrolled in infectious disease sites in Italy, so results are not directly generalizable to other geographical settings.

In conclusion, our data are important to our knowledge, as they provide for the first time, in a large cohort of PWH with suppressed viral load in the setting of modern ART, a description of newly developed comorbidities and reasons for switching ART. Our analysis also estimated the potential impact of these conditions on daily clinical management and identified potential determinants of ART switch in this PWH population. Specifically, new development of DP (especially in PWH treated with PI/b-based regimens) and a decline in eGFR of >10 mL/min/1.73m^2^ seem to be events that increase the probability of experiencing an ART change because of toxicity. In contrast, none of the considered NCDs seems to be associated with the probability of discontinuing ART because of simplification, and an increase in participants’ BMI was not at all associated with the probability of ART modification, nor among participants treated with INSTI-based regimens. Lifestyle modification interventions including diet, exercise and smoking cessation should be reinforced in patients who develop metabolic toxicity to avoid changing their ART regimen, which can potentially affect future drug options.

## Supplementary Material

dkaf137_Supplementary_Data

## Members of the ICONA Foundation Study Group

Board of Directors: A. d’Arminio Monforte (President), A. Antinori (Vice-President), S. Antinori, A. Castagna, R. Cauda, G. Di Perri, E. Girardi, R. Iardino, A. Lazzarin, G. C. Marchetti, C. Mussini, E. Quiros-Roldan, L. Sarmati, B. Suligoi, F. von Schloesser, P. Viale. Scientific Secretary: A. d’Arminio Monforte, A. Antinori, A. Castagna, F. Ceccherini-Silberstein, A. Cingolani, A. Cozzi-Lepri, A. Di Biagio, E. Girardi, A. Gori, S. Lo Caputo, G. Marchetti, F. Maggiolo, C. Mussini, M. Puoti, C. F. Perno, C. Torti. Steering Committee: A. Antinori, F. Bai, A. Bandera, S. Bonora, A. Calcagno, D. Canetti, A. Castagna, F. Ceccherini-Silberstein, A. Cervo, A. Cingolani, P. Cinque, A. Cozzi-Lepri, A. d’Arminio Monforte, A. Di Biagio, R. Gagliardini, A. Giacomelli, E. Girardi, N. Gianotti, A. Gori, G. Guaraldi, S. Lanini, G. Lapadula, M. Lichtner, A. Lai, S. Lo Caputo, G. Madeddu, F. Maggiolo, V. Malagnino, G. Marchetti, A. Mondi, V. Mazzotta, C. Mussini, S. Nozza, C. F. Perno, S. Piconi, C. Pinnetti, M. Puoti, E. Quiros Roldan, R. Rossotti, S. Rusconi, M. M. Santoro, A. Saracino, L. Sarmati, V. Spagnuolo, N. Squillace, V. Svicher, L. Taramasso, C. Torti, A. Vergori. Statistical and monitoring team: A. Cozzi-Lepri, S. De Benedittis, I. Fanti, N. Lentini, M. Muccio, R. Pastorino, A. Rodano', A. Tavelli. Community Advisory Board: M. Cernuschi, L. Cosmaro, A. Perziano, V. Calvino, D. Russo, M. Farinella, N. Policek, V. L. Del Negro. Biological Bank Inmi and San Paolo: M. Augello, S. Carrara, S. Graziano, G. Prota, S. Truffa, D. Vincenti, R. Rovito. Participating physicians and centres: Italy: A. Giacometti, A. Costantini, V. Barocci (Ancona); A. Saracino, C. Santoro, E. Milano (Bari); L. Comi, C. Suardi (Bergamo); P. Viale, L. Badia, S. Cretella (Bologna); E. M. Erne, A. Pieri (Bolzano); E. Quiros Roldan, E. Focà, C. Minardi (Brescia); B. Menzaghi, C. Abeli (Busto Arsizio); L. Chessa, F. Pes (Cagliari); P. Maggi, L. Alessio (Caserta); G. Nunnari, B. M. Celesia (Catania); J. Vecchiet, K. Falasca (Chieti); A. Pan, S. Dal Zoppo (Cremona); D. Segala (Ferrara); M. A. Di Pietro, C. Costa (Firenze); S. Lo Caputo, S. Ferrara (Foggia); M. Bassetti, E. Pontali, S. Blanchi, N. Bobbio, G. Mazzarello (Genova); M. Lichtner, L. Fondaco (Latina); S. Piconi, C. Molteni (Lecco); S. Rusconi, G. Canavesi (Legnano); G. Pellicanò (Messina); G. Marchetti, S. Antinori, G. Rizzardini, M. Puoti, A. Castagna, A. Bandera, V. Bono, M. V. Cossu, A. Giacomelli, R. Lolatto, M. C. Moioli, L. Pezzati, S. Diotallevi, C. Tincati (Milano); C. Mussini, M. Menozzi (Modena); P. Bonfanti, G. Lapadula (Monza); V. Sangiovanni, I. Gentile, V. Esposito, N. Coppola, F. M. Fusco, G. Di Filippo, V. Rizzo, N. Sangiovanni, S. Martini (Napoli); A. M. Cattelan, D. Leoni (Padova); A. Cascio, M. Trizzino (Palermo); D. Francisci, E. Schiaroli (Perugia); G. Parruti, F. Sozio (Pescara); D. Messeri, S. I. Bonelli (Pistoia); C. Lazzaretti, R. Corsini (Reggio Emilia); A. Antinori, R. Cauda, C. Mastroianni, L. Sarmati, A. Latini, A. Cingolani, I. Mastrorosa, S. Lamonica, M. Capozzi, M. Camici, M. Rivano Capparuccia, G. Iaiani, C. Stingone, L. Gianserra, J. Paulicelli, M. M. Plazzi, G. d’Ettore, M. Fusto (Roma); I. Coledan (Rovigo); G. Madeddu, A. De Vito (Sassari); M. Fabbiani, F. Montagnani (Siena); A. Franco, R. Fontana Del Vecchio (Siracusa); B. M. Pasticci, C. Di Giuli (Terni); G. C. Orofino, G. Calleri, G. Di Perri, S. Bonora, G. Accardo (Torino); C. Tascini, A. Londero (Udine); G. Battagin, S. Nicolè (Vicenza); G. Starnini, S. Dell’Isola (Viterbo).

## Transparency declarations

Outside of the submitted work: A.C.L. received research grants or contract from Icona Foundation Study (money paid to UCL) and European Union [Title: “EuCARE: European Cohorts of Patients and Schools to Advance Response to Epidemics.” Grant Agreement No 101046016 (money paid to UCL), Title “VIROMARKERS Consortium Agreement” Grant Agreement No 101194735 (money paid to UCL)]. L.T. attended advisory boards or served as consultant or received grants for conference participation from Gilead Sciences and ViiV Healthcare and research grants for her institution from Gilead Sciences. A.D.B. attended advisory boards or served as consultant or received grants for conference participation from Gilead Sciences and ViiV Healthcare and research grants for his institution from Gilead Sciences and ViiV Healthcare. G.L. has received speaking grants or consultancy fees from ViiV Healthcare, Pfizer srl, Insmed and Gilead srl over the past 5 years. V.M. declares payments and honoraria for lectures, presentations, speakers bureaus, manuscript writing or educational events by Pfizer. All other authors have no conflicts of interest to declare.

## Supplementary data

Figures S1–S4 and Tables S1–S5 are available as Supplementary data at *JAC* Online.
