# Supplementary material for: Selected comorbidities and the probability of ART switch in PWH with undetectable HIV-RNA: a retrospective analysis in Italy
Source: J Antimicrob Chemother. 2025 May 12;80(7):1849–59. doi: 10.1093/jac/dkaf137 (PMC12209853; doi:10.1093/jac/dkaf137)
Supplement: dkaf137_Supplementary_Data [file dkaf137_supplementary_data.zip › 4. Epic_Suppl_Fig1_JAC.docx]

PWH of Icona who:

1. had a period of >6 months with HIV-RNA ≤50copies/mL
2. the date of the 2^nd^ HIV-RNA ≤50 was after January 01, 2017
3. had ≥1 clinical visit post baseline and cases were identified

d2)

**Normal lipids at baseline**

***Stop due to simplification***

Cases N=684

Matched controls N=1,345

***Stop due to toxicity***

Cases N=252

Matched controls N=544

d3)

**eGFR>60 *ml/min/1.73m^2^* at baseline**

***Stop due to simplification***

Cases N=1,032

Matched controls N=1,960

***Stop due to toxicity***

Cases N=307

Matched controls N=632

d1)

**BMI≤25 kg/m^2^ at baseline**

***Stop due to simplification***

Cases N=680

Matched controls N=1,282

***Stop due to toxicity***

Cases N=219

Matched controls N=376

**Supplementary Figure 1.** Flow chart of PWH of Icona included in the analysis with breakdown by case-control study
