# Supplementary material for: Selected comorbidities and the probability of ART switch in PWH with undetectable HIV-RNA: a retrospective analysis in Italy
Source: J Antimicrob Chemother. 2025 May 12;80(7):1849–59. doi: 10.1093/jac/dkaf137 (PMC12209853; doi:10.1093/jac/dkaf137)
Supplement: dkaf137_Supplementary_Data [file dkaf137_supplementary_data.zip › 5. Epico_manus_Tab_Fig_Suppl_JAC[2].docx]

**Supplementary Figure S2.** Kaplan Meier estimate with 95% CI of the incidence of comorbidities after baseline in the sub-cohorts.

**A - BMI**

**B – DP**

**C- KD**

**Supplementary Table S1**. Characteristics of PLWH included in the case-control with exposure switch due to simplification and DP outcome

|  | **Incident dyslipidaemia** | | | |
| --- | --- | --- | --- | --- |
| **Characteristics at baseline^$^** | **Yes** | **No** | **p-value^*^** | **Total** |
|  | N= 218 | N= 1811 |  | N= 2029 |
| ***Gender, n(%)*** |  |  | 0.740 |  |
| Female | 46 (21.1%) | 400 (22.1%) |  | 446 (22.0%) |
| ***Mode of HIV Transmission, n(%)*** |  |  | 0.679 |  |
| PWID | 21 (9.6%) | 179 (10.0%) |  | 200 (9.9%) |
| Homosexual contacts | 105 (48.2%) | 817 (45.5%) |  | 922 (45.8%) |
| Heterosexual contacts | 78 (35.8%) | 706 (39.0%) |  | 784 (38.6%) |
| Other/Unknown | 14 (6.4%) | 93 (5.2%) |  | 107 (5.3%) |
| ***Nationality, n(%)*** |  |  | 0.004 |  |
| Not Italian | 21 (9.6%) | 315 (17.4%) |  | 336 (16.6%) |
| ***AIDS diagnosis, n(%)*** |  |  | 0.046 |  |
| Yes | 37 (17.0%) | 221 (12.2%) |  | 258 (12.7%) |
| ***CVD diagnosis, n(%)*** |  |  | 0.380 |  |
| Yes | 6 (2.8%) | 34 (1.9%) |  | 40 (2.0%) |
| ***HBsAg, n(%)*** |  |  | 0.543 |  |
| Negative | 195 (89.4%) | 1631 (90.1%) |  | 1826 (90.0%) |
| Positive | 2 (0.9%) | 31 (1.7%) |  | 33 (1.6%) |
| Not tested | 21 (9.6%) | 149 (8.2%) |  | 170 (8.4%) |
| ***HCVAb, n(%)*** |  |  | 0.920 |  |
| Negative | 175 (80.3%) | 1474 (81.4%) |  | 1649 (81.3%) |
| Positive | 26 (11.9%) | 206 (11.4%) |  | 232 (11.4%) |
| Not tested | 17 (7.8%) | 131 (7.2%) |  | 148 (7.3%) |
| ***Calendar year of baseline^**^*** |  |  | 0.013 |  |
| Median (IQR) | 2017 (2017, 2018) | 2018 (2017, 2019) | <.001 | 2018 (2017, 2019) |
| 2017 | 117 (53.7%) | 768 (42.4%) |  | 885 (43.6%) |
| 2018 | 58 (26.6%) | 513 (28.3%) |  | 571 (28.1%) |
| 2019 | 24 (11.0%) | 210 (11.6%) |  | 234 (11.5%) |
| 2020 | 9 (4.1%) | 111 (6.1%) |  | 120 (5.9%) |
| 2021 | 7 (3.2%) | 91 (5.0%) |  | 98 (4.8%) |
| 2022-2024 | 3 (1.4%) | 118 (6.5%) |  | 121 (6.0%) |
| ***Age, years*** |  |  | 0.011 |  |
| Median (IQR) | 47 (41, 54) | 45 (36, 53) |  | 46 (36, 53) |
| ***CD4 count, cells/mm3*** |  |  |  |  |
| Median (IQR) | 715 (498, 954) | 690 (515, 903) | 0.345 | 693 (515, 908) |
| <=200 cells/mmc | 6 (2.8%) | 39 (2.2%) | 0.571 | 45 (2.2%) |
| ***CD4 count nadir, cells/mm3*** |  |  | 0.465 |  |
| Median (IQR) | 306 (143, 434) | 306 (166, 450) |  | 306 (164, 448) |
| ***CD8 count, cells/mm3*** |  |  | 0.020 |  |
| Median (IQR) | 891 (659, 1173) | 821 (596, 1105) |  | 825 (602, 1115) |
| ***egfr (CKD_Epi formula), ml/min/1.73m^2^*** |  |  |  |  |
| Median (IQR) | 86.34 (71.13, 97.95) | 89.71 (77.07, 101.9) | 0.003 | 89.16 (76.30, 101.6) |
| Below 60, n(%) | 19 (8.7%) | 110 (6.1%) | 0.131 | 129 (6.4%) |
| ***Site geographical location, n(%)*** |  |  | 0.945 |  |
| North | 126 (57.8%) | 1028 (56.8%) |  | 1154 (56.9%) |
| Center | 74 (33.9%) | 624 (34.5%) |  | 698 (34.4%) |
| South | 18 (8.3%) | 159 (8.8%) |  | 177 (8.7%) |
| ***Diabetes, n(%)*** |  |  | 0.188 |  |
| Yes | 26 (11.9%) | 166 (9.2%) |  | 192 (9.5%) |
| ***Smoking, n(%)*** |  |  | 0.183 |  |
| No | 96 (44.0%) | 917 (50.6%) |  | 1013 (49.9%) |
| Yes | 93 (42.7%) | 685 (37.8%) |  | 778 (38.3%) |
| Unknown | 29 (13.3%) | 209 (11.5%) |  | 238 (11.7%) |
| ***Total cholesterol, mg/dL*** |  |  | <.001 |  |
| Median (IQR) | 181 (167, 210) | 177 (154, 201) |  | 177 (155, 203) |
| ***HDL cholesterol, mg/dL*** |  |  | <.001 |  |
| Median (IQR) | 44 (39, 51) | 51 (43, 61) |  | 50 (42, 60) |
| ***Use of statins, n(%)*** |  |  | 0.154 |  |
| Yes | 25 (11.5%) | 155 (8.6%) |  | 180 (8.9%) |
| ***Use of blood pressure lowering drugs, n(%)*** |  |  | 0.012 |  |
| Yes | 36 (16.5%) | 195 (10.8%) |  | 231 (11.4%) |
| ***Time from HIV diagnosis to baseline, months*** |  |  | 0.394 |  |
| Median (IQR) | 73 (31, 132) | 66 (27, 133) |  | 66 (28, 133) |
| ***Blood glucose, mg/dL*** |  |  | 0.012 |  |
| Median (IQR) | 88 (81, 98) | 86 (79, 94) |  | 86 (79, 95) |
| ***Type of regimen in episode, n(%)*** |  |  | 0.554 |  |
| Dual | 27 (12.4%) | 198 (11.3%) |  | 225 (11.4%) |
| Triple | 188 (86.6%) | 1535 (87.7%) |  | 1723 (87.6%) |
| Four or more drugs | 2 (0.9%) | 17 (1.0%) |  | 19 (1.0%) |
| PI-based | 45 (20.6%) | 391 (21.6%) | 0.748 | 436 (21.5%) |
| ***Education, n(%)*** |  |  | 0.141 |  |
| Primary school | 6 (2.8%) | 85 (4.7%) |  | 91 (4.5%) |
| Secondary school | 37 (17.0%) | 350 (19.3%) |  | 387 (19.1%) |
| College | 74 (33.9%) | 555 (30.6%) |  | 629 (31.0%) |
| University | 28 (12.8%) | 240 (13.3%) |  | 268 (13.2%) |
| Other/Unknown | 73 (33.5%) | 581 (32.1%) |  | 654 (32.2%) |
| ***Employment, n(%)*** |  |  | 0.885 |  |
| Unemployed | 21 (11.6%) | 196 (12.7%) |  | 217 (12.6%) |
| Employed | 103 (56.9%) | 793 (51.3%) |  | 896 (51.9%) |
| Self-employed | 32 (17.7%) | 283 (18.3%) |  | 315 (18.3%) |
| Occasional | 10 (5.5%) | 45 (2.9%) |  | 55 (3.2%) |
| Student | 2 (1.1%) | 78 (5.0%) |  | 80 (4.6%) |
| Retired | 3 (1.7%) | 49 (3.2%) |  | 52 (3.0%) |
| Invalid | 1 (0.6%) | 4 (0.3%) |  | 5 (0.3%) |
| Housewife | 5 (2.8%) | 53 (3.4%) |  | 58 (3.4%) |
| Other/unknown | 4 (2.2%) | 44 (2.8%) |  | 48 (2.8%) |
| ***Duration of VL suppression, months*** |  |  | 0.067 |  |
| Median (IQR) | 7.6 (6.5, 9.0) | 7.8 (6.7, 10.3) |  | 7.8 (6.6, 10.2) |
| ^$^Date of the switch (cases) or of last VL | | | | |
| ^*^Chi-square or Mann-Whitney test as appropriate | | | | |
| ^**^Sustanined VL suppression after Jan 2017 | | | | |

**Supplementary Figure S3.** Distribution of the main reasons for modification due to toxicity/intolerance (DP study)

**Supplementary Figure S4.** Class of the anchor drug used at baseline and after the modification due to simplification (only cases after DP exposure)

**Panel A**


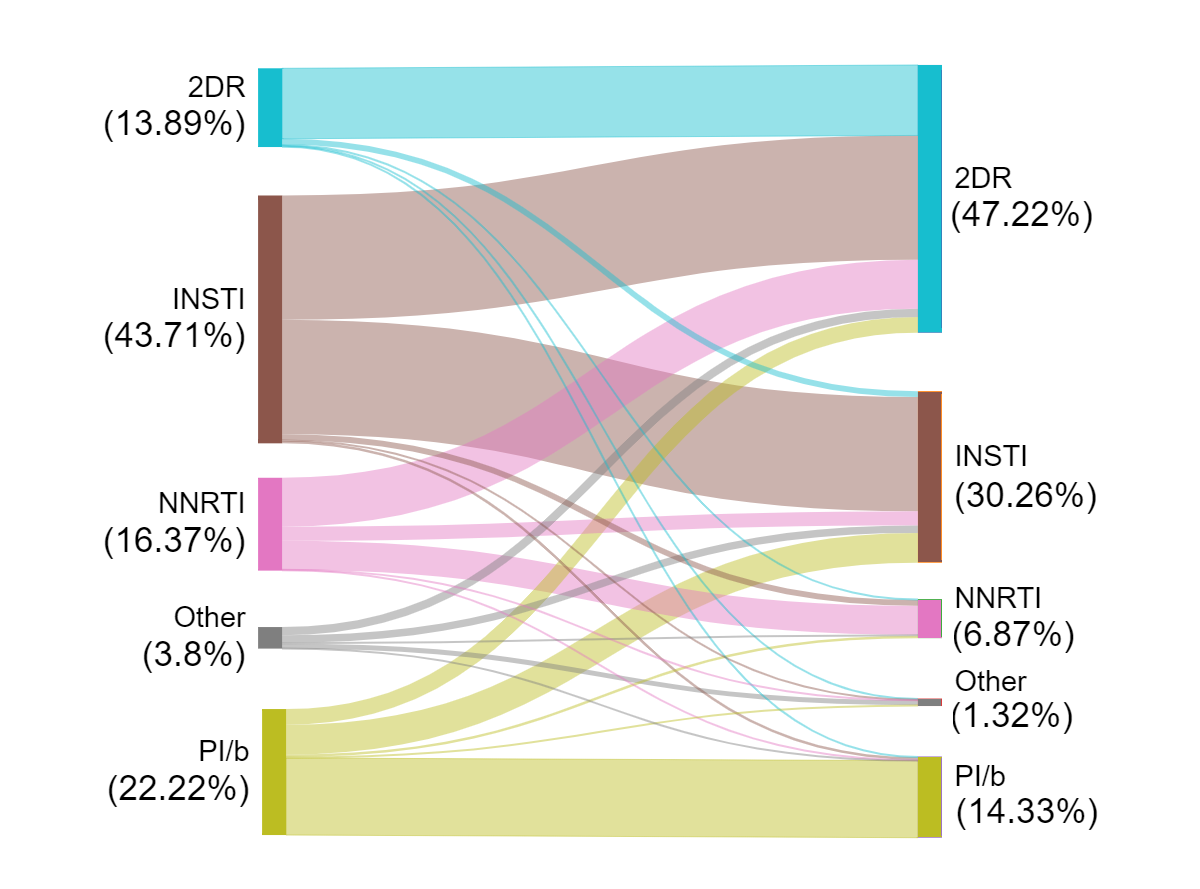


**Supplementary Figure S4.** Class of the anchor drug used at baseline and after the modification due to toxicity (only cases after DP exposure)

**Panel B**


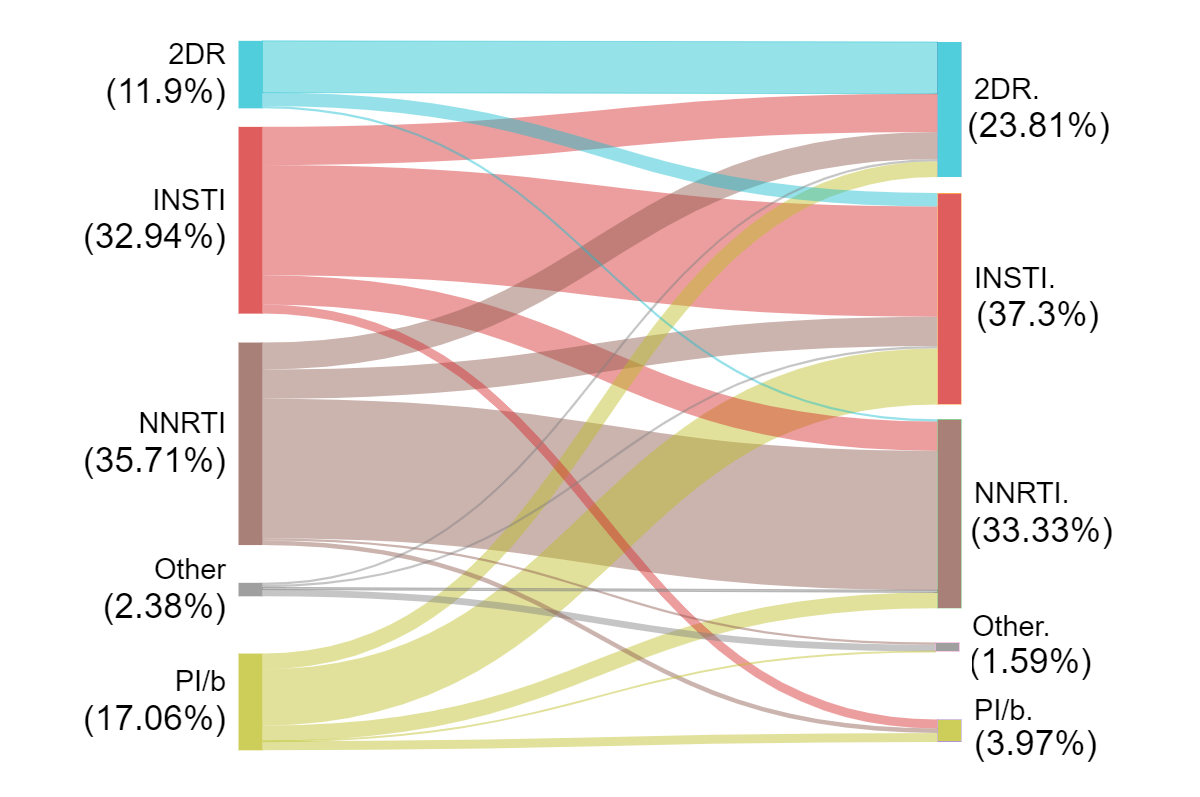


**Supplementary Table S2.** Odds ratio of ART modification due to simplification according to incident comorbidities from fitting a conditional logistic regression model with time-fixed confounding factors measured at baseline as well as type of regimen currently received

**Panel A**

|  | **Unadjusted and adjusted odds ratios  of discontinuation due to simplification** | | | | | |
| --- | --- | --- | --- | --- | --- | --- |
|  | **Cases** | **Controls** | **Unadjusted^&^** | | **Adjusted^&1,2,3^** | |
|  | **N(%)** | **N(%)** | **OR (95% CI)** | **p-value** | **aOR(95% CI)** | **p-value** |
| ***New onset of dyslipidaemia*** |  |  |  | 0.846 |  | 0.540 |
| No | 755 (90.0%) | 1246 (89.6%) | 1 |  | 1 |  |
| Yes | 84 (10.0%) | 145 (10.4%) | 0.97 (0.73, 1.29) |  | 0.88^1^ (0.58, 1.33) |  |
| ***New onset of eGFR<60 ml/min/1.73m^2^*** |  |  |  | 0.924 |  | 0.593 |
| No | 980 (95.0%) | 1869 (95.4%) | 1 |  | 1 |  |
| Yes | 52 (5.0%) | 91 (4.6%) | 1.02 (0.70, 1.48) |  | 0.90^2^ (0.60, 1.34) |  |
| ***Decline in eGFR>10 ml/min/1.73m^2^*** |  |  |  | 0.284 |  | 0.341 |
| No | 860 (83.3%) | 1661 (84.7%) | 1 |  | 1 |  |
| Yes | 172 (16.7%) | 299 (15.3%) | 1.12 (0.91, 1.40) |  | 1.12^2^ (0.89, 1.41) |  |
| ***New onset of BMI>26 Kg/m^2^*** |  |  |  | 0.699 |  | 0.458 |
| No | 640 (94.1%) | 1215 (94.8%) | 1 |  | 1 |  |
| Yes | 40 (5.9%) | 67 (5.2%) | 1.10 (0.67, 1.83) |  | 1.35^3^ (0.61, 2.99) |  |
| ***Increase in BMI>3 Kg/m^2^*** |  |  |  | 0.936 |  | 0.455 |
| No | 250 (92.9%) | 411 (92.2%) | 1 |  | 1 |  |
| Yes | 19 (7.1%) | 35 (7.8%) | 1.04 (0.41, 2.66) |  | 1.38^3^ (0.59, 3.23) |  |
| ^&^matched for sex at birth and age | | | | | | |
| ^1^Adjusted for year of baseline, age, sex at birth, obesity, alcohol use, nationality, CD4 base, AIDS and current regimen  ^2^Adjusted for year of baseline, age, diabetes, sex at birth, CD4 base, AIDS and current regimen  ^3^Adjusted for year of baseline, age, sex at birth, nationality, CD4 base, AIDS and current regimen | | | | | | |
|  | | | | | | |

**Supplementary Table S2.** Odds ratio of ART modification due to toxicity/intolerance according to incident comorbidities from fitting a conditional logistic regression model with time-fixed confounding factors measured at baseline as well as type of regimen currently received

**Panel B**

|  | **Unadjusted and adjusted odds ratios  of discontinuation due to toxicity/intolerance** | | | | | |
| --- | --- | --- | --- | --- | --- | --- |
|  | **Cases** | **Controls** | **Unadjusted^&^** | | **Adjusted^&1,2,3^** | |
|  | **N(%)** | **N(%)** | **OR (95% CI)** | **p-value** | **aOR(95% CI)** | **p-value** |
| ***New onset of dyslipidaemia*** |  |  |  | 0.002 |  | 0.016 |
| No | 219 (86.9%) | 510 (93.8%) | 1 |  | 1 |  |
| Yes | 33 (13.1%) | 34 (6.3%) | 2.43 (1.37, 4.32) |  | 2.46 (1.18, 5.15) |  |
| ***New onset of eGFR<60 ml/min/1.73m^2^*** |  |  |  | 0.350 |  | 0.217 |
| No | 296 (96.4%) | 605 (95.7%) | 1 |  | 1 |  |
| Yes | 11 (3.6%) | 27 (4.3%) | 0.67 (0.30, 1.54) |  | 0.58 (0.25, 1.38) |  |
| ***Decline in eGFR>10 ml/min/1.73m^2^*** |  |  |  | 0.033 |  | 0.103 |
| No | 257 (83.7%) | 565 (89.4%) | 1 |  | 1 |  |
| Yes | 50 (16.3%) | 67 (10.6%) | 1.59 (1.04, 2.44) |  | 1.45 (0.93, 2.28) |  |
| ***New onset of BMI>26 Kg/m^2^*** |  |  |  | 0.585 |  | 0.703 |
| No | 211 (96.3%) | 362 (96.3%) | 1 |  | 1 |  |
| Yes | 8 (3.7%) | 14 (3.7%) | 0.77 (0.30, 1.98) |  | 0.82 (0.31, 2.22) |  |
| ***Increase in BMI>3 Kg/m^2^*** |  |  |  | 0.866 |  | 0.590 |
| No | 45 (88.2%) | 94 (92.2%) | 1 |  | 1 |  |
| Yes | 6 (11.8%) | 8 (7.8%) | 1.19 (0.16, 8.60) |  | 1.42 (0.40, 5.03) |  |
| ^&^matched for sex at birth and age | | | | | | |
| ^1^Adjusted for year of baseline, age, sex, obesity, alcohol use, nationality, CD4 base, AIDS and current regimen  ^2^Adjusted for year of baseline, age, diabetes, sex, CD4 base, AIDS and current regimen  ^3^Adjusted for year of baseline, age, sex, nationality, CD4 base, AIDS and current regimen | | | | | | |

**Supplementary Table S3.** Odds ratio of ART modification due to toxicity/intolerance according to incident eGFR from fitting a conditional logistic regression model with time-fixed confounding factors measured at baseline after restricting to participants receiving INSTI at baseline

|  | **Unadjusted and adjusted odds ratios  of discontinuation due to toxicity - restricted to INSTI** | | | | | |
| --- | --- | --- | --- | --- | --- | --- |
|  | **Cases** | **Controls** | **Unadjusted^&^** | | **Adjusted^&*^** | |
|  | **N(%)** | **N(%)** | **OR (95% CI)** | **p-value** | **aOR(95% CI)** | **p-value** |
| ***New onset of egfr<60*** |  |  |  | 0.180 |  | 0.076 |
| No | 296 (96.4%) | 301 (98.0%) | 1 |  | 1 |  |
| Yes | 11 (3.6%) | 6 (2.0%) | 3.11 (0.59, 16.32) |  | 5.33 (0.84, 33.93) |  |
| ***Decline in egfr>10*** |  |  |  | 0.062 |  | 0.051 |
| No | 257 (83.7%) | 605 (95.7%) | 1 |  | 1 |  |
| Yes | 50 (16.3%) | 27 (4.3%) | 2.66 (0.95, 7.45) |  | 2.84 (1.00, 8.07) |  |
| ^&^matched for sex at birth and age | | | | | | |
| ^*^matched for sex at birth and age and adjusted for year of baseline, AIDS and CD4 count | | | | | | |

**Supplementary Table S4.** Odds ratio of ART modification due to toxicity/intolerance according to incident eGFR (using different definitions) from fitting a conditional logistic regression model with time-fixed confounding factors measured at baseline

|  | **Unadjusted and adjusted odds ratios  of discontinuation due to toxicity** | | | | | |
| --- | --- | --- | --- | --- | --- | --- |
|  | **Cases** | **Controls** | **Unadjusted^&^** | | **Adjusted^&*^** | |
|  | **N(%)** | **N(%)** | **OR (95% CI)** | **p-value** | **aOR(95% CI)** | **p-value** |
| ***New onset of eGFR<60 – single value*** |  |  |  | 0.350 |  | 0.325 |
| No | 296 (96.4%) | 605 (95.7%) | 1 |  | 1 |  |
| Yes | 11 (3.6%) | 27 (4.3%) | 0.67 (0.30, 1.54) |  | 0.66 (0.28, 1.52) |  |
| ***New onset of eGFR<60- 2 consecutive values*** |  |  |  | 0.787 |  | 0.969 |
| No | 257 (83.7%) | 565 (89.4%) | 1 |  | 1 |  |
| Yes | 50 (16.3%) | 67 (10.6%) | 1.15 (0.41, 3.28) |  | 0.98 (0.34, 2.85) |  |
| ***Decline in egfr>10*** |  |  |  | 0.033 |  | 0.064 |
| No | 301 (98.0%) | 621 (98.3%) | 1 |  | 1 |  |
| Yes | 6 (2.0%) | 11 (1.7%) | 1.59 (1.04, 2.44) |  | 1.51 (0.98, 2.32) |  |
| ^&^matched for sex at birth and age | | | | | | |
| ^*^matched for sex at birth and age and adjusted for year of baseline, AIDS and CD4 count | | | | | | |

**Supplementary Table S5.** Odds ratio of ART modification due to simplification according to incident BMI (using the alternative WHO definition) from fitting a conditional logistic regression model with time-fixed confounding factors measured at baseline

**Panel A**

|  | **Unadjusted and adjusted odds ratios  of discontinuation due to simplification** | | | | | |
| --- | --- | --- | --- | --- | --- | --- |
|  | **Cases** | **Controls** | **Unadjusted^&^** | | **Adjusted^&*^** | |
|  | **N(%)** | **N(%)** | **OR (95% CI)** | **p-value** | **aOR(95% CI)** | **p-value** |
| ***New onset of bmi>=265*** |  |  |  | 0.464 |  | 0.373 |
| No | 557 (92.8%) | 1056 (93.2%) | 1 |  | 1 |  |
| Yes | 43 (7.2%) | 77 (6.8%) | 0.82 (0.49, 1.38) |  | 0.79 (0.47, 1.33) |  |
| ^&^matched for sex at birth and age | | | | | | |
| ^*^matched for sex at birth and age and adjusted for year of baseline, AIDS and CD4 count | | | | | | |

**Supplementary Table S5.** Odds ratio of ART modification due to toxicity/intolerance according to incident BMI (using the alternative WHO definition) from fitting a conditional logistic regression model with time-fixed confounding factors measured at baseline

**Panel B**

|  | **Unadjusted and adjusted odds ratios  of discontinuation due to toxicity** | | | | | |
| --- | --- | --- | --- | --- | --- | --- |
|  | **Cases** | **Controls** | **Unadjusted^&^** | | **Adjusted^&*^** | |
|  | **N(%)** | **N(%)** | **OR (95% CI)** | **p-value** | **aOR(95% CI)** | **p-value** |
| ***New onset of bmi>=25 (WHO definition)*** |  |  |  | 0.576 |  | 0.557 |
| No | 189 (95.5%) | 314 (94.6%) | 1 |  | 1 |  |
| Yes | 9 (4.5%) | 18 (5.4%) | 0.72 (0.23, 2.24) |  | 0.71 (0.23, 2.22) |  |
| ^&^matched for sex at birth and age | | | | | | |
| ^*^matched for sex at birth and age and adjusted for year of baseline, AIDS and CD4 count | | | | | | |
